# Spatial and Social Organization in a Burrow-Dwelling Lizard (*Phrynocephalus vlangalii*) from China

**DOI:** 10.1371/journal.pone.0041130

**Published:** 2012-07-23

**Authors:** Yin Qi, Daniel W. A. Noble, Jinzhong Fu, Martin J. Whiting

**Affiliations:** 1 Chengdu Institute of Biology, Chinese Academy of Sciences, Chengdu, Sichuan, China; 2 Department of Biological Sciences, Macquarie University, Sydney, New South Wales, Australia; 3 Department of Integrative Biology, University of Guelph, Guelph, Ontario, Canada; Institut Pluridisciplinaire Hubert Curien, France

## Abstract

Shared ecological resources such as burrow complexes can set the stage for social groupings and the evolution of more complex social behavior such as parental care. Paternity testing is increasingly revealing cases of kin-based groupings, and lizards may be a good system to inform on the early evolution of sociality. We examined spatial and social organization in the lizard *Phrynocephalus vlangalii* from China and tested genetic relatedness (based on eight microsatellite DNA loci) between offspring and parents that shared burrow complexes. Adult males and females had similar spatial patterns: they overlapped most with members of the opposite sex and least with their own sex. Males in better body condition overlapped with more females, and both sexes showed high site fidelity. Most lizards used a single burrow, but some individuals used two or three burrows. While high site fidelity is consistent with sociality in lizards, juveniles did not preferentially share burrows with parents, and we documented only a few cases of parent–offspring associations through burrow sharing. We suggest that *P. vlangalii* conforms to a classical polygynous mating system in which the burrow forms the core of the male's territory and may be offered as an important resource for females, but this remains to be determined.

## Introduction

Sociality (long-term stable groups with overlapping generations) in vertebrates has traditionally been thought to be restricted primarily to birds and mammals, which clearly demonstrate long-term cohesive groups [1], [2]. Complex social behavior is predicted to evolve when related and/or unrelated groups of individuals merge [2], [3], setting the scene for the evolution of cooperative and altruistic behavior, parental care and group foraging, all commonly observed in birds and mammals [1], [2]. Recent work, however, in other ‘less’ social vertebrates, such as lizards, has revealed simple forms of sociality [4], [5], [6], [7]. Lizards exhibit less complex forms of sociality compared with mammals and birds, involving aggregations of kin and non-kin groups [5], [8] that are sometimes associated with rudimentary forms of parental care [9]. For example, desert night lizards (*Xantusia vigilis*) aggregate under logs in groups of 2–18 individuals [5]. Juvenile aggregations with adults tend to be genetically related and most individuals belong to the same nuclear family. Such aggregations result from delayed dispersal of juvenile lizards, generating simple kin-based sociality in *Xantusia* [5]. Similar kin-based associations have been recorded in multiple species in the *Egernia-Liopholis* clade of Australian skinks, which typically form nuclear families [4], [10]. More recently, the Australian lizard *Liopholis kintorei*, which lives in burrows excavated in sandy deserts, has been documented to live in nuclear families in which group members ‘cooperate’ to maintain the burrow system [7]. These systems provide a unique opportunity to explore the early stages and evolution of sociality. However, the paucity of ecological and genetic studies in the vast majority of lizard clades precludes statements regarding the general form of sociality in lizards.

Parental care is a particularly prevalent form of social behavior in birds and mammals; however, it is rare among lizards [1], [11]. Although maternal care in lizards has evolved multiple times, it generally takes on a rudimentary form [1]. For example, Taiwanese long-tailed skinks (*Mabuya longicaudata*) show simple forms of maternal care where females brood and actively defend clutches of eggs from egg-eating snakes [6], [12]. Similar egg brooding behaviors have been recorded in the North American skinks of the *Plestiodon* [formally *Eumeces*] complex [13] and pythons [14]. However, studies in live-bearing (viviparous) skink species suggest that maternal care can manifest itself in more complex forms [9]. In *Egernia whitii*, offspring born to aggressive females have higher survival compared with offspring born to less aggressive females and this may be the result of decreased infanticide [9]. Similarly, the black rock skink, *Egernia saxatilis*, lives in family groups in which the presence of a parent significantly reduces the likelihood of infanticide [10], [15]. More complex forms of maternal care in reptiles may be associated with the evolution of viviparity because it provides greater opportunity for interaction between parents and offspring [5].

Defending key resources (territoriality) necessary for both adult and offspring survival may be an important stepping-stone for the evolution of parental care [16]. For example, defending a burrow or crevice that is limited may provide a direct fitness benefit to adults and aggressive behaviors necessary for resource defense may cross functional contexts and promote the protection of offspring from conspecifics [12], [17]. Alternatively, sheltering sites within a territory may provide protection from predators or stochastic climatic conditions [18]. Systems where all of the above criteria are met may provide an opportunity to test for the presence of parental care and provide important insight into the diversity of social systems in lizards.

**Figure 1:**
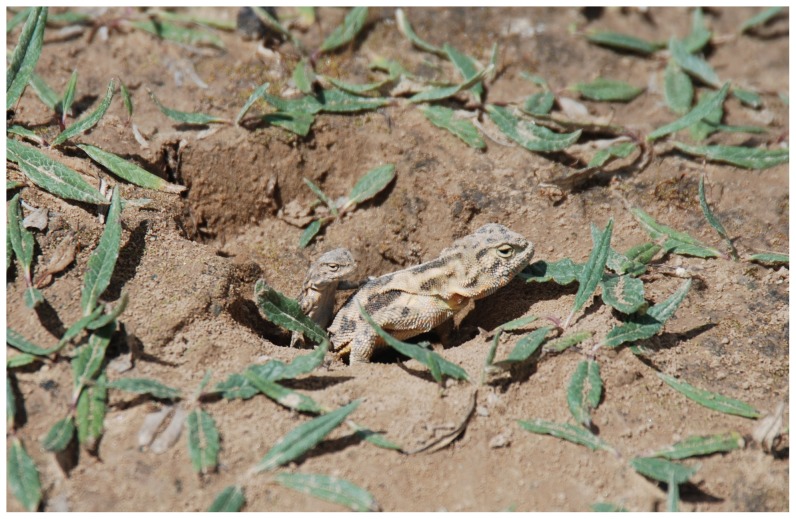
Female *Phrynocaphalus vlangalii* in a burrow with a young juvenile.

The Qinghai Toad-headed Agama (*Phrynocephalus vlangalii*; Figure 1) is a high-elevation, viviparous lizard found in the northern part of the Tibetan Plateau [19]. Both males and females are highly aggressive and use complex tail displays during social interactions [20]. Tail curling may function in establishing male social rank by signaling individual body condition [21]. Lizards also have a tail-tip badge that is sexually dimorphic: orange in females and black in males. *Phrynocephalus vlangalii* excavate burrows to approximately 70 cm in loose sand [22] and these burrows are essential for over-winter survival because temperatures can drop to well below zero during the winter months (−10.3±1.9°C to −2.4±1.46°C; Monthly average temperatures from November–March). Our observations of a population of *P. vlangalii* in Xiamen Nature Reserve revealed that adults and offspring sometimes occupied excavated burrows together (Figure 1). Here, we combine two years of data on the spatial and social organization of *P. vlangalii* with molecular estimation of individual relatedness to test the hypothesis that offspring found within adult burrows are part of a parent-offspring relationship and suggestive of sociality. We also explore morphological differences between the sexes because of its implications in understanding lizard mating systems and territoriality. Testing the parent-offspring hypothesis is a first step in identifying whether kin-based sociality and parental care may be present in this taxonomically differentiated group of lizards. We first address patterns of burrow use by adult males and females and then test whether adult lizards may be sharing burrows with their offspring during the winter, a critical period of their life.

## Results

### Morphology

We tested for significant differences between males and females in morphological traits that typically correlate with lizard mating systems. When year was controlled for, mean male snout-vent length (SVL) was significantly greater than mean female SVL (Table 1; ANCOVA; Sex: F_1, 168_ = 11.85, p<0.001; year (covariate): F_1, 168_ = 4.702, p = 0.03; Sex*Year: F_1, 168_ = 2.35, p = 0.13). When both SVL and year were controlled for, mean male head length was significantly greater than mean female head length (Table 1; ANCOVA; Sex: F_1, 167_ = 27.45, p<0.001; SVL (covariate): F_1, 167_ = 91.48, p<0.001; Year (covariate): F_1, 167_ = 9.04, p<0.01; Sex*Year: F_1, 167_ = 2.01, p = 0.16). Mean male head width was significantly greater than mean female head width (Table 1; ANCOVA; Sex: F_1, 167_ = 7.60, p<0.01; SVL (covariate): F_1, 167_ = 90.57, p<0.001; year (covariate): F_1, 167_ = 128.64, p<0.001; Sex*Year: F_1, 167_ = 1.85, p = 0.18).

**Table 1:** Mean ±SE (N) snout-vent length (mm), head length (mm) and head width (mm) of male and female *P. vlangalii* captured in 2009 and 2010 in Xiamen Nature Reserve.

	2009		2010		Combined (2009–2010)	
	Male	Female	Male	Female	Male	Female
Snout vent length (SVL)	57.29±0.40 (35)	56.47±0.52 (41)	57.11±0.49 (35)	54.68±0.52 (61)	57.20±0.31 (70)	55.40±0.38 (102)
Head length	16.76±0.15 (35)	16.44±0.18 (41)	16.52±0.23 (35)	15.46±0.15 (61)	16.64±0.13 (70)	15.85±0.13 (102)
Head width	15.30±0.12 (35)	15.42±0.21 (41)	13.94±0.14 (35)	13.45±0.12 (61)	14.62±0.13 (70)	14.24±0.15 (102)

The combined (2009–2010) is the mean of the morphological measurements for 2009 and 2010 combined. Parentheses indicate sample sizes.

### Use of burrows and space

**Figure 2:**
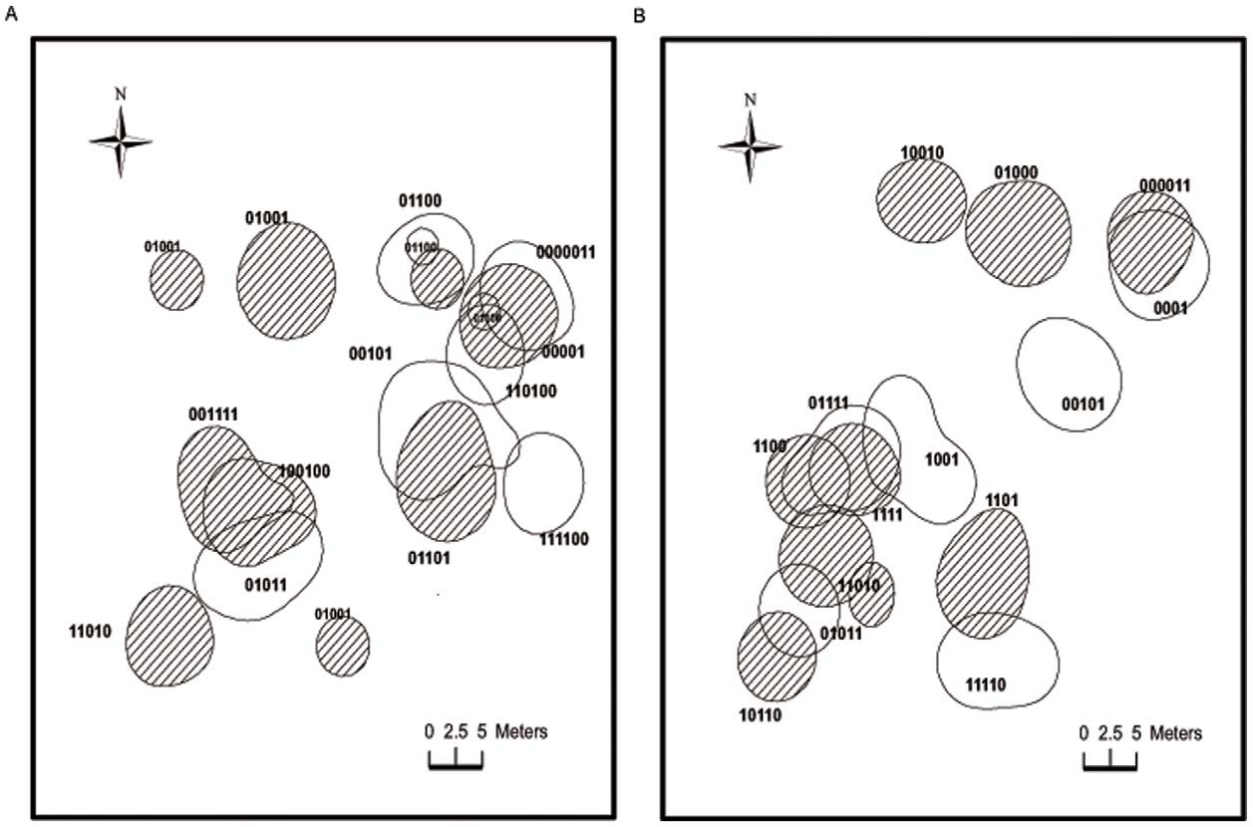
Spatial distribution of *Phrynocephalus vlangalii*. Spatial distribution and 75% Kernel home ranges of a subset of male (n = 12) and female (n = 14) lizards collected during A) 2009 and B) 2010. Cross-hatching refers to females, empty spaces to males. Males (01011 and 00101) and female (11010) were marked in 2009 and recorded at the same position in 2010. Some individuals have two spatially separate areas because the Kernel method calculates areas of intensive use (see Methods).

Fifty-three (87%) male lizards were classified as residents (used the same burrow for ten or more days) during the breeding season and 10 (12.5%) males were located in both years. The average number of burrows each male occupied was 1.41±0.11 (n = 29 from 2010; Table 2). Of these, 66% occupied one burrow, 27% occupied two burrows and 7% occupied three burrows. Each male was re-sighted with a mean frequency of 6.88±0.49 (n = 53) over 18.43±0.82 days (n = 53 during our 30-day census period). We also classified 62 (77%) female lizards as residents during the breeding season. The average number of burrows each female occupied was 1.30±0.09 (n = 30; Table 2). Of these, 71% occupied one burrow and 29% occupied two burrows. Each female was re-sighted with a mean frequency of 6.46±0.40 (n = 62) over 18.02±0.62 days (n = 62) during our 30-day census period (Figure 2, Table 2). We recaptured 11 females in 2010.

**Table 2:** The number of unique lizards captured in 2009 and 2010.

	2009		2010		Combined (2009–2010)	
	Male	Female	Male	Female	Male	Female
Number of lizards marked	35	41	35	61	70	102
Number of lizards resighted	28	32	33	49	61	81
Mark-recapture rate	0.80	0.78	0.94	0.80	0.87	0.79
Lizards captured: 2009 and 2010	-	-	10	11	10	11
Number of residents	25	24	28	38	53	62
Proportion of residents	0.89	0.72	0.85	0.73	0.87	0.77
Number of sightings	7.20±0.78	7.58±0.65	8.18±0.81	5.66±0.38	6.88±0.49	6.46±0.40
	(25)	(24)	(28)	(38)	(53)	(62)
Home range area (m^2^)	48.60±14.60	30.20±10.9	38.20±11.70	14.56±5.58	43.75±9.29	32.01±8.79
	(8)	(10)	(7)	(9)	(15)	(19)
Maximum distance moved (m)	11.99±4.09	10.61±2.39	15.54±4.86	5.25±1.70	13.76±3.09	8.41±1.66
	(7)	(10)	(7)	(7)	(14)	(17)
Number of burrows occupied	-	-	1.41±0.11	1.29±0.07	1.30±0.13	1.30±0.09
			(29)	(38)	(20)	(30)

We scored lizards as residents if they used the same burrow for 10 or more days (see text). Summary statistics (means ±1SE) are reported for the spatial data. Sample sizes are indicated in parentheses. Home range area was estimated using the minimum convex polygon.

Male home range area averaged 43.75±9.29 m^2^, while females averaged 32.01±8.79 m^2^ (Table 2; 2009–2010 combined). The mean maximum distance a male moved from his burrow was 13.8±3.09 m (n = 14) while females moved a mean maximum distance of 8.41±1.66 m (n = 17), and was not significantly different between the sexes (W = 152, p = 0.19; Figure 2). Home range area was not significantly different between the sexes and was not related to SVL (ANCOVA: Sex: F_1, 32_ = 3.29, p = 0.08; SVL (covariate): F_1, 32_ = 1.09, p = 0.30). Body condition was not significantly correlated with home range area in males (r = 0.05, p = 0.86, n = 15). Male home range overlapped with an average of 1.27±0.33 other males (n = 15, range = 1–4) and 1.67±0.43 females (n = 15, range = 1–5). Female home range overlapped with an average of 1.00±0.29 females, (n = 19, range = 1–4) and 1.84±0.53 males (n = 19, range = 1–8) (Table 3). Male body condition was significantly positively correlated with the number of females overlapped (r_s_ = 0.61, p = 0.02, n = 15) and with overlap pressure on females (r_s_ = 0.65, p = 0.01, n = 15).

**Table 3:** Measures of home range overlap in *P. vlangalii* for adult males with at least 11 sightings (n = 14) and females with at least 8 sightings (n = 13).

	Number overlapped (mean ±SE (n))	Overlap pressure (mean ± SE (n))
♂ on ♀	1.67±0.43 (15)	0.08±0.03 (15)
♂ on ♂	1.27±0.33 (15)	0.11±0.04 (15)
♀ on ♂	1.84±0.53 (19)	0.10±0.04 (19)
♀ on ♀	1.00±0.29 (19)	0.11±0.03 (19)

### Burrow sharing and relatedness in adults and offspring

A total of 97 lizards were found in 54 (68%) of the 80 burrows we excavated. Seventy-one (73%) of these lizards were offspring, seven (7%) were adult males and 19 (20%) were adult females. Offspring were found on their own or with other young in 28 burrows while they were found with adult males and/or females in 15 burrows. A single adult female was found with 1–3 offspring in eight instances while a single adult male was found with 1–5 offspring on five occasions. An adult male and female were found together in the same burrow only once, along with one baby. Ten females and one male were collected from burrows with no other individuals.

**Figure 3:**
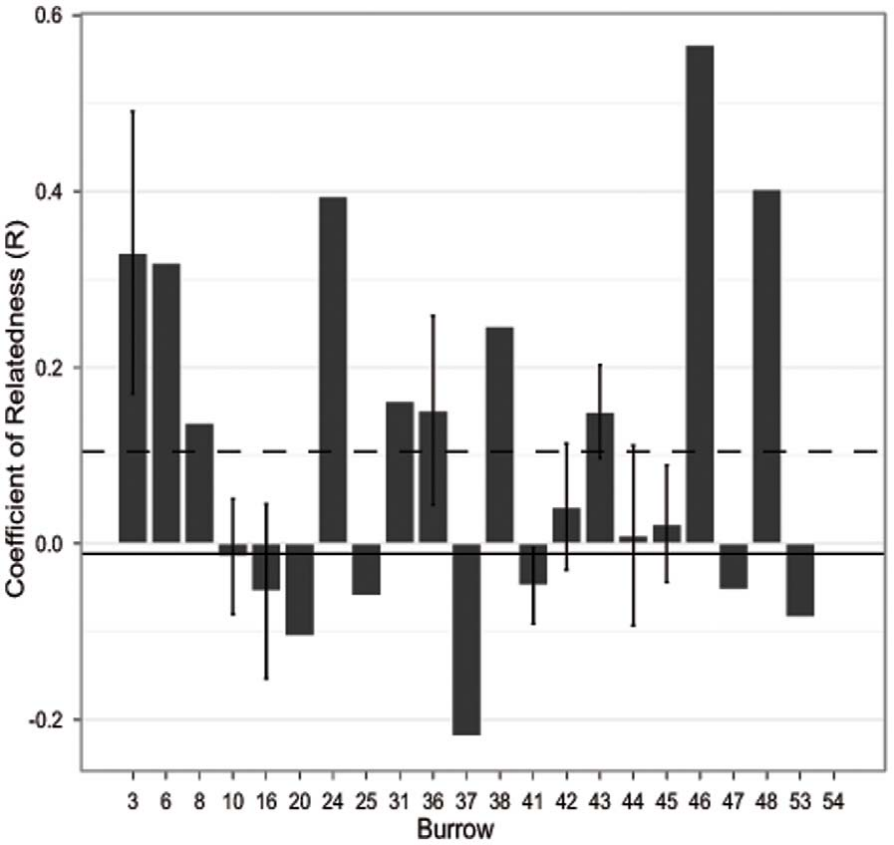
Relatedness of individuals within burrows. Mean (±1SE) relatedness coefficient, R [43], for 22 burrows containing two or more individual *P. vlangalii*. The dashed line indicates the overall mean burrow relatedness while the solid line is the population mean relatedness.

Coefficient of relatedness (R) estimates ranged from −0.57 to 0.77 among all possible pairs of individuals in the sample. The population level mean relatedness estimate was −0.012±0.003. Of the 54 burrows, three (5.5%; Burrows 3, 24, 46) showed evidence of significant parent-offspring relationships. Burrow 3 contained three offspring and a single adult female. Two of the offspring showed significant parent-offspring relationships with the adult female (R = 0.71–0.77, p<0.001), while the third baby was unrelated. Burrow 24 contained one adult female and a single baby that were significantly related at the parent-offspring level (R = 0.39, p = 0.001) while burrow 46 contained one adult male and a single related offspring (R = 0.54, p<0.001). In all of these cases the adults and offspring shared alleles at 100% of their loci. In total, seven (32%) of the 22 burrows with multiple individuals had higher relatedness than the population burrow mean (Figure 3).

We identified 30 burrow groups that contained from 1–10 (mean ± SE = 3.23±0.40) burrows within 2 m of each other. We compared parent-offspring relationships among the individuals found in nearby burrows because we know that some lizards will occupy multiple burrows and the maximum distance between these burrows is very small (2 m). We identified one more parent-offspring relationship (in addition to the burrows identified as having significant parent-offspring relationships above) in a single burrow group. Burrow group 9 contained an adult female in burrow 20 and her offspring in burrow 19 where the primary hypothesis of parent-offspring relatedness could not be rejected (R = 0.41, p = 0.0023). The parent and offspring in this instance also shared alleles at 100% of their loci. Burrows 20 and 19 were 1.5 m apart.

## Discussion

Our study revealed remarkably similar patterns in burrow occupancy for males and females. Both sexes showed relatively high site fidelity within a season and typically occupied a single burrow although some (ca. 30%) occupied a second burrow and a small proportion (ca. 7%) a third burrow. Individuals from both sexes were also resighted at almost the same frequency (close to 7 sightings). However, males appeared to move more and were seen as much as 14 m from their burrows while females moved a maximum of just over 8 m from their burrows; males also had larger home ranges although high variance meant this relationship was marginally non-significant. These differences were not explained by body size and likely reflect sex-specific life history tactics. Both sexes also had some spatial overlap in their home ranges. In terms of the number of individuals overlapping in space, spatial overlap was greatest between the sexes, compared to within the sexes. That is, males were more likely to overlap with females while females were more likely to overlap with males than other females. Also, males in better body condition overlapped with more females. In terms of overlap pressure–the amount of space shared with another individual, there were no clear patterns and the values were similar for both sexes albeit that a relatively small part of their home range was shared space. Finally, offspring did not show a strong preference for sharing burrows with adult genetic relatives and when they did, only a third of the cases showed higher than average levels of relatedness with only three burrows supporting parent-offspring relationships.

*Phrynocephalus vlangalii* conforms to the typical pattern of space use seen in territorial lizards, which is an exclusive core area that overlaps with members of the opposite sex [18], [23]. Males display frequently, are aggressive, and the core of their territory is their burrow entrance. This corresponds to a resource-specific site defence [18], which has also been documented in the burrow-dwelling barking gecko (*Ptenopus g. garrulus*; [24]). However, whether females choose mates based on individual quality or some aspect of burrow quality is unknown. Interestingly, females are also highly aggressive, and maintain territories that largely exclude other females, which is the case for many iguanids and agamids (reviewed in [23]). As a consequence, females do not aggregate and males are unable to defend multiple mates and do not appear to mate guard individual females. In resource-based polygynous systems, males that monopolise high quality resources have higher fitness than males in low quality habitats [25]. We did not see any evidence of variation in territory quality because the study area appeared uniform in vegetation structure and food availability. However, males in better body condition were able to overlap more females, suggesting that variance in reproductive success is linked to the control of space. There is also the intriguing prospect that females may benefit from occupying male burrows, which males actively defend. However, there were more burrows than lizards, allowing females the option of avoiding males, and females are capable of digging their own burrows (YQ, unpubl. data). The extent to which females use self-constructed burrows versus other burrows is not known. Finally, male *P. vlangalii* were larger in body size and had larger head dimensions compared to females, which is typical in polygynous mating systems [26].

The social organisation for this species, coupled with the shared use of burrows, raises the possibility of a cryptic kin-based social system. However, for convincing evidence of parent-offspring associations we predicted frequent burrow sharing between parents and offspring or at the very least, that they would occupy neighbouring burrow systems just a few metres away. Given that adult lizards defend burrows, a key refuge for young lizards, it could be adaptive for offspring to associate with their parents for several reasons. First, a simple association with a parent could be sufficient to prevent infanticide or aggression from an unrelated adult [10]. Second, parents may even directly intervene in the case of a predation threat. For example, female long-tailed skinks (*Eutropis longicaudata*) actively deter snake predators from their nests [12] and an adult King's skink (*Egernia kingii*) was once observed chasing off a snake that ventured too close to the family refuge [27]. Although we did find evidence that some burrows contained parent-offspring relationships, our observations largely did not support this prediction. First, there were many instances of either solitary, or multiple, offspring in burrows where there was no adult present. Second, the relatively low number of offspring that we did find with at least one parent could be explained by chance given that young lizards are born within the home range of their mother. Even if these few individuals delayed dispersal to remain in the presence of a parent, this is likely to be a weak selective force for the evolution of sociality. Delayed dispersal and social groupings are being documented in more lizard species [4], [5], with the realisation that aggregations of individuals may be the product of social selection. The role of key resources as a potential trigger for the evolution of sociality is as yet poorly understood, but could be a fruitful line of future research.

In summary, we first focused on burrow and space use to establish the potential for social groupings that might influence sociality through parent-offspring associations. Burrows are a key resource necessary for offspring survival, particularly over winter, and young juveniles in their first year of life seem to be dependent on abandoned burrows or in rare cases, the burrows of their parents. Patterns in spatial overlap largely conformed to predictions of sexual selection theory for males in a polygynous mating system: higher overlap with females than with other males [23]. Female *P. vlangalii* also tended to avoid each other, were observed to be aggressive towards one another, and were more likely to overlap in space with males than females. We did not find evidence of strong spatial and/or pair bonds between males and females that might set the stage for sociality. We suggest that *P. vlangalii* conforms to a classical polygynous mating system tied to resource defence, although detailed studies are required to test the potential value of male-defended burrows to females, and to determine the degree of polygyny.

## Methods

### Ethics statement

All handling and processing of lizards followed approved protocols from the Chengdu Institute of Biology of the Chinese Academy of Sciences. We also followed the ABS (Animal Behavior Society)/ASAB (Association for the Study of Animal Behaviour) “Guidelines for the treatment of animals in behavioural research and teaching”. The Chengdu Institute of Biology approved this work and all fieldwork was approved by the Forestry Department of the Sichuan Provincial Government and the Management Office of the Zoige Nature Reserve.

### Study area

We conducted fieldwork at Xiamen Conservation Station in the Zoige Wetland Nature Reserve, southwestern China (33°43′25″N, 102°29′4″E; elev. ca. 3464 m) during the breeding season (May–June of 2009 and 2010). *Phyrnocephalus vlangalii* occur at a density of 0.19 lizards/m^2^ on large, sparsely vegetated sand dunes that are patchy across the landscape [22]. The vegetation on and around these sand dunes is predominantly composed of the grasses *Kobresia humilis*, *Kobresia prattii* and *Elymus natans* and a shrub, *Salix sclerophylla*.

### Morphological measurements and the use of burrows and space

A 0.2 ha plot (50×40 m) was marked out and divided into 20 quadrants, each 10×10 m [28]. Lizards in the plot were caught by noosing or by pitfall traps. Pitfall traps were simply small holes (ca. 30 cm deep×15 cm diameter) that were dug at the entrance to their burrows and which we filled in after catching the lizard or at the end of the day if we were unsuccessful. All individuals greater than 45 mm snout-vent-length (SVL) were considered sexually mature and were processed immediately following capture. Although we do not know when individuals become sexually mature in this population the smallest size we observed females reproducing was 45 mm SVL (YQ, unpubl. data) and all the males we collected were greater than 50 mm SVL. Lizards were marked permanently by toe-clipping and given a unique color code on their dorsum using non-toxic acrylic paint to facilitate later identification. Sex was determined by checking for hemipenal bulges. Mass was measured with a Pesola**®** spring scale to the nearest 0.1 g. SVL (snout-vent length), head length (snout to neck length) and head width (distance between the posterior end of the right and left mandible) were measured using digital calipers to the nearest 0.1 mm. Body condition was estimated using the residuals from the regression of log body mass on log SVL [29].

We conducted censuses between 09:00–16:30 h during which lizards were located by slowly walking through the study area four times a day. The census route took approximately 60 min and we scan sampled for lizards at each quadrat for a total of three minutes before moving onto the next quadrat. Upon sighting a lizard, we recorded their locations, and when possible, the locations of their burrows, using x-y coordinates which we later mapped using ArcGis (9.3) software. We started at a different quadrat each time to minimize any potential bias in the number of sightings per individual. We used only sightings of lizards that were separated by at least 2 h. In total, we marked 76 lizards (35 ♂ and 41 ♀) from 11 May to 11 June 2009, and 96 lizards (35 ♂ and 61 ♀) from 18 May to 16 June 2010. Lizards captured in both years were only included once (2009) in any morphological and spatial analyses.

We scored lizards as residents if they used the same burrow for ten or more days, which is a criterion that has been used in other studies [28], [30], [31]. We estimated home range area and maximum movement distance from a burrow for each lizard using Hawth's Tools (www.spatialecology.com), a plug-in for ArcGIS, by calculating the minimum convex polygons (MCP), Kernel and distance between points. MCP has been suggested to be good at estimating the total area of an individual's home range, but is influenced by the number of individual relocations [32]. The Kernel method, however, estimates the utilization distribution within the home range and has been shown to overestimate home range area [32], [33], [34]. Row and Blouin-Demers [34] suggested that MCP and Kernel methods should be used simultaneously in calculating individual home range area. In our analysis, MCP was used to estimate the area of the Kernel, while Kernel estimation was used to indicate the utilization distribution.

We used the method of Smith [35] to determine the minimum number of sightings needed for home range estimation by running a series of regressions with MCP home range as the dependent variable. We started with all individuals having at least three sightings (47 males and 41 females), the number of sightings necessary for inclusion was increased until there was no longer a statistically significant relationship between home range size and the number of sightings. For males, 11 or more sightings were necessary for MCP home range estimation (sightings ≤10, r = 0.55, n = 18, p = 0.01), while for females only nine or more sightings were required (sightings ≤8, r = 0.40, n = 19, p = 0.08). For the Kernel method, the grid size was set to 1 m and the smoothing factor set to 3. After comparing the two methods, we determined that the 75% Kernel best described lizard home range area because it assigned similar areas with those assigned by the MCP method.

The amount of overlap between home range areas was calculated with ArcGis (9.3) software. For each individual the number of overlapping males and females was calculated as well as overlap pressure between individuals of the same and opposite sex [36]. We calculated overlap pressure by adding all the areas that other individuals shared with the focal individual and then dividing this sum by the focal individual's home range size. This results in a score from 0-n where n is the degree of overlap pressure. We counted the number of overlapping individuals and calculated overlap pressure because both of them are standard estimations of overlap that have been used in other studies [31], .

We analyzed the differences in SVL, head length and head width between the sexes using ANCOVA. For the SVL comparison, we include sex as the main factor and year as a covariate. For head length and head width comparisons, we included sex as the main factor with both the year and SVL as covariates. To control for possible differences between years, we also included an interaction between sex and year in all models. In some cases differences between the sexes varied by year (although they were not significant) and we separately provided means and standard errors for each sex by year and combined (Table 1). We tested for differences in home range area between the sexes using ANCOVA, controlling for SVL. For males, we used both body condition and SVL as a measurement of individual quality, while for females, we only used SVL because we could not account for any influence of reproductive condition which may affect body mass. Spearman rank correlations were used to examine the relationships between body condition, SVL, home range size and home range overlap. All data were analyzed using R (version 2.14 for Windows, freely available at http://www.r-project.org).

### Burrow sharing and relatedness in adults and offspring

We excavated 80 burrows during the hibernation period (November 2010) that were marked in September to test whether juveniles over-wintered with their parents. We chose the over-wintering period to address this question because a burrow is essential for over-wintering survival and this would be the most important time for parents to provide burrows for offspring. All lizards collected in their burrows were toe-clipped (for DNA), measured for body size, sexed, and relocated to the Chengdu Institute of Biology for further behavioral study.

Genomic DNA was extracted from 97 tissue samples collected from lizards found within burrows using the DNeasy Tissue extraction kit (QIAGEN) according to the manufacturer's protocol. We used eight microsatellite DNA loci (Phr27, Phr79, Phr63, Phr160, PVMS12, PVMS18, PVMS35 and PVMS38) for which primers were already developed [38], [39]. PCR amplification was performed in 25 uL reaction volumes containing 1 uL of extracted DNA, 12.5 uL PCR mix (TransGen), 1 uL of each primer (10 pmol uL^−1^) and 9.5 uL of dd H_2_O. Forward primers of Phr27, PVMS18 and PVMS38 were labeled with FAM fluorescein, forward primers of Phr79 and PVMS35 were labeled with HEX fluorescein, and forward primers of Phr63, PVMS12 and Phr160 were labeled with TAM fluorescein. Reactions took place in a thermocycler (Mastercycler pro, Eppendorf) with an initial denaturation of 94°C for 5 minutes, then 30 cycles at 94°C for 30 s, T_a_ for 30 s, and 72°C for 1 min followed by 72°C for 10 min. The primer specific annealing temperatures (T_a_) can be found in [39] and [38]. The fluorescent-labeled PCR products were pooled and alleles were separated using an ABI PRISM 3730 capillary sequencer (Applied Biosystems) and scored using Genemapper *vers*. 1.95.

We used Kingroup *vers*. 2.0 (Konovalov *et al*. 2004), which makes use of the method developed by Queller and Goodnight [40] and Goodnight and Queller [41], to calculate pairwise relatedness and test hypothesized relationships among individuals found within the same burrows (or nearby burrows). The program uses likelihood-based methods on genotypic data when both the maternal and paternal alleles are unknown by calculating the average likelihood values for all possible assumptions about the maternal and paternal origin of alleles [41]. The product of the individual likelihoods are then taken over all the loci and support for the primary vs. null hypothesis is evaluated using a likelihood ratio between the two hypotheses [41]. The statistical significance of the hypothesized relationship is calculated by simulating pairs of individuals of known relationship, where alleles are drawn at random from the population allele frequencies for one individual and then according to the null hypothesis to be tested for the second individual. The program proceeds by creating a large number of simulated pairs that are related according to the null hypothesis. The value of the likelihood ratio, which excludes 95% of these pairs in the simulated likelihood ratio distribution, is then used to test statistical significance between pairs of individuals [41].

We tested whether adult lizards found within the same burrows as offspring were parents of those offspring. *Phrynocephalus vlangalii* is known to occupy from 1–3 distinct burrows (this study). Therefore, we also tested the relationship between offspring and adults found in separate burrows if these burrows were within 2 m of each other. A distance of 2 m was chosen because this is the largest distance between burrows used by a single individual. We differentiate between these two analyses by referring to within-burrow comparisons ( = burrows) and between-burrow comparisons ( = burrow groups). In all hypothesis tests likelihood calculations were done by simulating 10,000 pairs based on the population allele frequencies from the 97 individuals collected in the study. Any pair where a parent-offspring relationship was supported was checked manually to ensure that the individuals shared alleles at all eight loci.

## References

[1] Reynolds JD, Goodwin NB, Freckleton RP (2002). Evolutionary transitions in parental care and live bearing in vertebrates.. P R Soc B.

[2] Alexander RD (1974). The evolution of social behavior.. Annu Rev Ecol Syst.

[3] Earley RL, Dugatkin LA, Westneat DF, Fox CW (2010). Behavior in groups..

[4] Chapple D (2003). Ecology, life history and behavior in the Australian scincid lizard *Egernia* with comments on the evolution of complex sociality in lizards.. Herpetol Monogr.

[5] Davis AR, Corl A, Surget-Groba Y, Sinervo B (2011). Convergent evolution of kin-based sociality in a lizard.. P R Soc B.

[6] Huang WS (2006). Parental care in the long-tailed skink, *Mabuya longicaudata*, on a tropical Asian island.. Anim Behav.

[7] McAlpin S, Duckett P, Stow A (2011). Lizards cooperatively tunnel to construct a long-term home for family members.. PLoS One.

[8] Chapple DG, Scott Keogh J (2006). Group structure and stability in social aggregations of White's skink, *Egernia whitii*.. Ethology.

[9] Sinn DL, While GM, Wapstra E (2008). Maternal care in a social lizard: links between female aggression and offspring fitness.. Anim Behav.

[10] O'Connor DE, Shine R (2004). Parental care protects against infanticide in the lizard *Egernia saxatilis*.. Anim Behav.

[11] Shine R, Gans C, Huey RB (1988). Parental care in reptiles..

[12] Huang W-S, Pike DA (2011). Climate change impacts on fitness depend on nesting habitat in lizards.. Funct Ecol.

[13] Vitt LJ, Cooper WE (1989). Maternal Carein Skinks (*Eumeces*).. J Herpetol.

[14] Lourdais O, Hoffman TCM, DeNardo DF (2007). Maternal brooding in the children's python (*Antaresia childreni*) promotes egg water balance.. J Comp Physiol B.

[15] O'connor D, Shine R (2003). Lizards in ‘nuclear families’: a novel reptilian social system in *Egernia saxatilis* (Scincidae).. Mol Ecol.

[16] Brown JL, Morales V, Summers K (2010). A key ecological trait drove the evolution of biparental care and monogamy in an amphibian.. Am Nat.

[17] While GM, Sinn DL, Wapstra E (2009). Female aggression predicts mode of paternity acquisition in a social lizard.. P R Soc B.

[18] Stamps JA, Gans C, Tinkle DW (1977). Social behavior and spacing patterns in lizards..

[19] Zhao KT, Zhao EM, Zhao KT, Zhou KY (1999). *Phrynocephalus* kaup..

[20] Qi Y, Li S, Suo L, Li H, Wang Y (2011). An ethogram of the toad-headed lizard *Phrynocephalus vlangalii* during the breeding season.. Asian Herpetol Res.

[21] Qi Y, Wan HF, Gu HJ, Wang Y (2011). Do displays and badges function in establishing the social structure of male toad-headed lizards, *Phrynocephalus vlangalii*?. J Ethol.

[22] Wu P, Zeng Z, Wang Y, Zhu B (2005). A new method for investigating *Phrynocephalus vlangalii* population density.. Chinese J Ecol.

[23] Stamps JA, Huey RB, Pianka ER, Schoener TW (1983). Sexual selection, sexual dimorphism, and territoriality..

[24] Hibbitts TJ, Whiting MJ, Stuart-Fox DM (2007). Shouting the odds: vocalization signals status in a lizard.. Behav Ecol Sociobiol.

[25] Emlen S, Oring L (1977). Ecology, sexual selection, and the evolution of mating systems.. Science.

[26] McCoy JK, Baird TA, Fox SF, Fox SF, McCoy JK, Baird TA (2003). Sexual Selection, Social Behavior, and the Environmental Potential for Polygyny..

[27] Masters C, Shine R (2003). Sociality in lizards: family structure in free-living King's Skinks *Egernia kingii* from southwestern Australia.. Aust Zool.

[28] Aragon P, Lopez P, Martin J (2001). Seasonal changes in activity and spatial and social relationships of the Iberian rock lizard, *Lacerta monticola*.. Can J Zool.

[29] Green AJ (2001). Mass/length residuals: Measures of body condition or generators of spurious results?. Ecology.

[30] Stapley J, Keogh JS (2005). Behavioral syndromes influence mating systems: floater pairs of a lizard have heavier offspring.. Beha Ecol.

[31] Morrison SF, Keogh JS, Scott IAW (2002). Molecular determination of paternity in a natural population of the multiply mating polygynous lizard *Eulamprus heatwolei*.. Mol Ecol.

[32] Rose B (1982). Lizard home ranges: methodology and functions.. J Herpetol.

[33] Worton BJ (1987). A review of models of home range for animal movement.. Ecol Model.

[34] Row JR, Blouin-Demers G, Fox SF (2006). Kernels are not accurate estimators of home-range size for herpetofauna.. Copeia.

[35] Smith G (1995). Home range size, overlap, and individual growth in the lizard, *Sceloporus virgatus*.. Acta Oecol.

[36] Smith JM (1977). Parental investment: a prospective analysis.. Anim Behav.

[37] Reaney LT, Whiting MJ (2003). Are female tree agamas (*Acanthocercus atricollis atricollis*) turned on by males or resources? (vol 15, pg 30, 2003).. Ethol Ecol Evo.

[38] Zhan A, Fu J (2009). Microsatellite DNA markers for three toad-headed lizard species (*Phrynocephalus vlangalii*, *P. przewalskii* and *P. guttatus*).. Mol Ecol Resour.

[39] Urquhart J, Bi K, Gozdzik A, Fu JZ (2005). Isolation and characterization of microsatellite DNA loci in the toad-headed lizards, *Phrynocephalus przewalskii* complex.. Mol Ecol Notes.

[40] Queller DC, Goodnight KF (1989). Estimating relatedness using genetic markers.. Evolution.

[41] Goodnight K, Queller D (1999). Computer software for performing likelihood tests of pedigree relationship using genetic markers.. Mol Ecol.

